# “Collateral beauty.” Experiences and needs of professionals caring for parents continuing pregnancy after a life-limiting prenatal diagnosis: A grounded theory study

**DOI:** 10.1177/02692163241255509

**Published:** 2024-05-30

**Authors:** Konstanze Wiesner, Kerstin Hein, Gian Domenico Borasio, Monika Führer

**Affiliations:** Center for Pediatric Palliative Care, Dr von Hauner Children’s Hospital, LMU University Hospital, LMU Munich, Munich, Germany

**Keywords:** Perinatal palliative care, personal growth, palliative care, perinatal palliative care program, grounded theory

## Abstract

**Background:**

Caring for parents continuing pregnancy after learning about a severe life-limiting condition in their unborn is challenging. Most existing studies focus on affected families, whereas research on the subjective experience of care professionals is scarce.

**Aim:**

We aimed to (1) explore experiences and needs of involved care professionals, (2) obtain information about existing care structures, and (3) identify requirements for a structured perinatal palliative care program.

**Design:**

Grounded Theory study using theoretical sampling. Data was collected by semi-structured interviews and analyzed following the principles of grounded theory coding and situational analysis.

**Setting:**

A total of 18 professionals from 12 different services in Munich and surroundings participated in the study: 8 physicians, 3 midwives, 2 nurses, 1 each pregnancy counselor, grief counselor, chaplain, clinical psychologist, and undertaker.

**Results:**

Several organizations provide support for affected parents, but inter-institutional communication is scarce. Due to the lack of a dedicated perinatal palliative care program, professionals make immense and partly unpaid efforts to support concerned parents. Providers experience “collateral beauty” in their work despite all the suffering and grief. This includes the development of a humble attitude and feelings of gratitude toward life, the feeling of having a meaningful task and professional as well as personal growth. Requirements for a structured perinatal palliative care program include: fostering peer support, ensuring regular supervision, and enhancing interdisciplinary exchange.

**Conclusions:**

Perinatal palliative care demands a high level of personal engagement but is experienced as highly rewarding by care professionals.

**What is already known about the topic:**

- Perinatal palliative care is supposed to support affected families in a holistic way, including medical and psycho-social care as well as spiritual guiding.
- Yet there is little research addressing the experiences, expectations, and perceptions of the professionals providing care in a perinatal palliative situation.

**What this paper adds:**

- Delivering perinatal palliative care to parents choosing to continue pregnancy after a life-limiting prenatal diagnosis is experienced as emotionally challenging and professionally demanding, but at the same time highly rewarding.
- Inadequate care structures require that professionals make an immense and partly unpaid effort to support concerned parents.

**Implication for practice, theory, or policy**

- Care professionals need structures who emphasize reflection and foster insight about ethical, professional and personal themes arising through perinatal palliative care.
- Interdisciplinary exchange, for example, through intervision groups, workshops, and conferences, ought to be enhanced.

## Introduction

Perinatal palliative care teams provide holistic support to families who choose to continue with pregnancy despite the diagnosis of a life-limiting disease in their unborn child as part of their broader mandate.^1^ This includes medical, psychosocial, and spiritual care^2^ delivered by an interdisciplinary team.^3–7^ A wide range of professions and services are involved in the care of affected families,^8^ depending on local conditions, parents’ needs and diagnosis of the child,^9^ including obstetricians/gynecologists, prenatal diagnosticians, neonatologists, pediatricians, palliative care specialists, genetic counselors, midwives, nurses, psychologists, social workers, and spiritual assistants.^9–14^

Previous studies consistently report that affected parents criticize the poor communication skills and inadequate accompaniment provided by care professionals. Many parents have the impression that professionals do not understand their decision to continue pregnancy.^15–17^ There is a repeated demand for better education and training of care providers regarding perinatal palliative care and communication.^18–20^ To improve the quality of perinatal palliative care programs, the professionals’ needs and experiences should be taken into account.^8,21,22^

Caring for affected parents in perinatal palliative care represents a challenging and emotionally demanding task, and care professionals are at risk for suffering from moral distress, burnout, posttraumatic stress disorder.^19, 22–27^ compassion fatigue,^28^ grief,^29^ and existential suffering.^19^ Therefore, a structured perinatal palliative care program should support care professionals to cope with their burdensome work.^9,12,19,20,24^

In contrast to the broad body of research about the parents’ experiences and needs, there is little research investigating the experiences, expectations, and perceptions of the professionals providing perinatal care for families continuing with pregnancy after learning about a life-limiting disease in their unborn child.^9,20^ Our study aims to close this gap. In addition, by collecting information about existing care structures and cooperation networks, we seek to identify requirements for a structured perinatal palliative care program.

*Note: We wish to point out that this article does not concern, and is completely neutral about, the decision for or against termination of pregnancy. All quotes reflect the opinions of the interviewees and do not necessarily reflect the opinions of the researchers.*

## Methods

### Study design

We conducted expert interviews with care providers from Munich and surroundings who care for parents continuing pregnancy after a life-limiting prenatal diagnosis using a Grounded Theory approach.^30^ We considered Grounded Theory as a suitable approach to explore the subjective perspective of care providers involved in perinatal palliative care and to generate grounded and thus meaningful hypothesis about the topic.

Grounded Theory comprises a set of qualitative methods for gathering and analyzing data to construct theories that emerge from data.^30^ Grounded Theory has evolved since its beginnings resulting in the existence of several variations of the method. We conducted our study in concordance to the principles of the constructivist Grounded Theory, which acknowledges the role of the subjective perspective of researchers, the mutual creation of knowledge, and the interpretative nature of results.^30^ In addition, we used elements of the Situational Analysis,^31^ which corresponds to the latest development of Grounded Theory. Situational analysis acknowledges the complexity and diversity of a particular situation and seeks to generate “thick descriptions”^32^ about its elements and relations by mapping the situation, social arenas, and relevant positions in prevailing discourses.^[Bibr bibr10-02692163241255509] 31 [Bibr bibr33-02692163241255509]^ Given the heterogeneous, cross-disciplinary and cross-institutional organization of perinatal palliative care and the poorly developed interconnection between care providers,^10,33^ we considered Situational Analysis as a suitable complementary tool to grasp the complexity of the field.

The reporting of the method follows the *Consolidated criteria for reporting qualitative research* (COREQ).^34^ The study protocol and materials were approved by the ethics committee of the LMU Munich University Hospital (no: 21-0174).

### Sampling

Participants were selected by theoretical sampling.^30^ The purpose of theoretical sampling is to seek pertinent data to develop the emerging theory. It starts with an initial sampling, for which the researcher establishes sampling criteria before entering the field.^30^ Sampling criteria for the initial sampling were defined based on previous studies about the topic,^10,33^ which identified the key role of the following caregivers: gynecologists, prenatal diagnosticians, midwives, neonatologists, psychosocial counselors, and undertakers. Therefore, eligibility criteria in the initial sampling were: (1) care provider of one of the identified key professions working in Munich or surrounding areas, (2) experience with perinatal palliative care (at least one case), (3) sufficient German language skills, (4) and written informed consent.

The subsequent sampling strategy was guided by the ongoing analysis of data and no longer restricted to the key care professions. It followed the eligibility criteria of the initial sampling in that it considered professionals in Munich and surroundings with experience in perinatal palliative care, sufficient language skills, and written informed consent.

### Recruitment

We identified the participants of the initial sample through gatekeepers: a physician and midwife and a spiritual assistant, both experts in perinatal palliative care. We also contacted a network of professionals of Munich and surroundings dealing with the topic of pre- and perinatal deaths.

The subsequent recruitment was guided by the ongoing analysis of data. Gatekeepers and other study participants helped to identify and recruit further participants (snowball sampling). All potential participants were contacted by K.W. They received detailed written information about the goals, methods, and data protection aspects before signing up for the study. Sampling was concluded after reaching theoretical saturation, which means that no further theoretical insights were expected by gathering more data.^30^

### Data collection

Data were collected using semi-structured expert interviews^35,36^ conducted by K.W. between April 2021 and February 2022. We considered our participants to be experts who possess special knowledge or expertise about their professional field,^35,36^ which would enable us to reconstruct the provision of care of parents continuing with pregnancy after learning about the diagnosis of their child.

The Supplemental interview guideline was developed by K.W. and a psychologist with expertise in qualitative research (K.H.) and discussed within the context of a qualitative research course at the university. The main topics of the interview were the experience with families continuing with pregnancy after prenatal diagnosis, the experience regarding existing structures of care, and the collaboration with other care providers.

Due to the pandemic, most interviews took place online via RED Connect, which is a secure peer-to-peer videocall-tool. Only few were carried out face to face. The average duration of an interview was approximately 90 min (range: 60–120 min). All interviews were carried out by K.W. Interviews were preceded by a short questionnaire about sociodemographic data. All participants gave written informed consent to their participation in the study. Interviews were audiotaped, transcribed verbatim, and pseudonymized. Audio files and transcripts were stored on institutional, password-protected servers. Only the research team had access to the data.

The Supplemental interview guideline is available from the first author (K.W.). To maintain confidentiality, the participants’ informed consent does not allow us to share the complete interviews with third parties.

### Data analysis

Transcripts were analyzed by KW following the principles of Grounded Theory as outlined by Charmaz.^30^ Charmaz divides the process of analysis into initial coding, focused coding, and theoretical coding. Initial coding creates first data-driven codes by means of the constant comparison method.^30,37^ During initial coding, the researcher remains open to exploring different analytic possibilities. In comparison, focused coding is directive and selective. It centers on the most significant codes that explain larger amounts of data. During theoretical coding, the researcher explores the relationship between the categories to develop an integrated theoretical framework. KW also wrote memos throughout the whole research process.^30^ Coding was supported by the software MAXQDA 20. Codes were discussed in research meetings between all authors.

In addition, all authors compiled a social arenas map, in accordance with the principles of Situational Analysis,^31^ which provides an overview of the collective actors involved in perinatal palliative care and the relationships between them.

## Results

We approached 40 care professionals, of whom 18 accepted to participate in the study. Main reasons for not wanting to participate includes lack of time and not enough experience in the topic. Participants came from 12 different services. One prenatal diagnostician was interviewed twice, because new questions developed after analyzing the interview. Most participants were female (*n* = 16) and had additional qualifications in grief and trauma support and palliative care. A detailed description of the sample is given in Table 1.

**Table 1.** Characteristics of participants (*n* = 18).

Characteristics	Participants
Gender	
Female	16
Male	2
Age (years)	
Mean	47.5
Range	25–73
Profession	
Physician	Two neonatologists, one pediatric palliative care specialist, five gynecologists/obstetricians (three with special training in prenatal diagnostics)
Midwife	3
Pediatric nurse	2
Spiritual assistant	1
Undertaker	1
Clinical psychologist	1
Pregnancy counselor	1
Grief counselor	1
Professional experience (years)	
Median	17.3
Range	3–44
Number of cases	
Median	13.4
Range	1 – 30
Employment	
Employee	10
Self - employed	6
Combination of both	2
Denomination	
Catholic	12
Protestant	1
No religion	3
No information	2

Despite differing trainings and fields of work, professionals shared similar experiences in caring for affected parents.

We constructed four main categories from data: Framing the situation, management of perinatal palliative care, perceptions of families and „Collateral beauty.” Collateral beauty represents the core concept. It refers to the professional growth, gratitude, and humbleness care professionals experience during the provision of perinatal palliative care. Caregivers cope with the challenging character of the situation in that they reframe it as a positive experience (framing the situation). At the same time, they are confronted with unclear structures of care, which they have to manage (management of perinatal palliative care). Provision of care is conceived in accordance with the perceived needs of families (perception of families).

### Framing the situation

Professionals highlighted that not only the health care system but also society are profit and performance driven. They stated that death and dying are marginalized and pregnancy is perceived as an event without room for error. Interviewees sensed that in society everything, especially the unborn, must be perfect. Therefore, a dying unborn child would not fit the system.

*“And also this horizon of expectations, this expectation of the good, healthy, perfect child.” (PD3 – prenatal diagnostician)*

In this context, professionals stressed that parents saw termination of pregnancy as a quick and sterile solution. Participants thought that most people in Germany believe that physicians tend to favor termination of pregnancy. Yet, most of our interviewees showed great understanding for parents carrying their pregnancy to term.

*“I also like to accompany such families [who carry on the pregnancy], because we are often confronted with families who say, I want a termination as soon as possible, and I find it very enriching for my work that people take their time, that they get involved, that the path emerges as they walk.” (PD1-prenatal diagnostician)*

Participants indicated that when the death of the child is inevitable, there is no need for hectic actions, but space for humanity, compassion, and communality between parents and caregivers. Emotions, discussions about ethics and shared values were allowed. Interviewees thought that this makes perinatal palliative care a unique and highly personalized experience for families and professionals – representing a counterpart to a highly technical medicine.

*“The beauty of it is that . . . that there are no invasive, aggressive interventions where anyone has to decide that any life is terminated, neither the parents nor . . . nor us. (. . .) It’s whole, this picture, even though it’s so sad, yes. So nobody is interfering or doing anything. And that’s the beauty of it.” (P – Clinical Psychologist)*

### The management of perinatal palliative care

**Figure 1.**
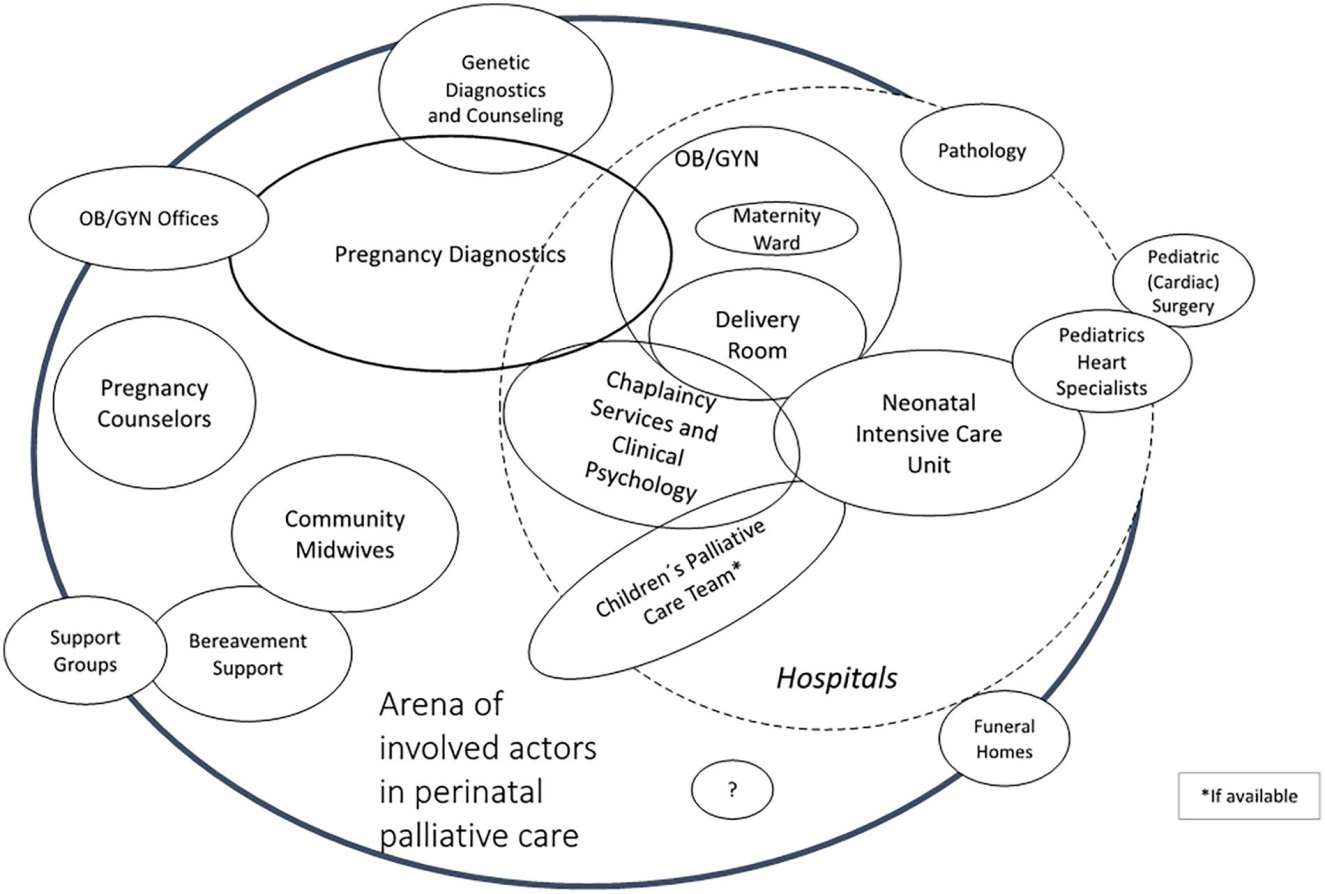
Social Arena map according to Situational Analysis. The map shows all potentially involved actors in a perinatal palliative care setting, as identified through our interviews. The central circle denotes the core actors, while the size of the circles surrounding each actor indicates their importance in care provision and frequency of involvement.

We found numerous institutions in Munich and the surrounding area that offer perinatal palliative care to some extent, including small private practices, two university hospitals, other hospitals, counseling centers and even funeral homes (see Figure 1). However, interviews indicated that these institutions have little interaction with each other, and in some cases even compete against each other for patients and resources within the health care system. Consequently, many professionals were unaware of the existence of other organizations offering perinatal palliative care.

Our findings suggest that, among physicians, neonatologists, and gynecologists/obstetricians with training in prenatal diagnostics were especially involved. Midwives played a role from early pregnancy to post-partum visits. Nurses were involved after birth in intensive care units and specialized pediatric palliative home services. Psychosocial support encompassed pregnancy counselors, spiritual assistants, psychologists, and grief counsellors. Somewhat unexpectedly, data analysis also suggested a significant role of specialized undertakers.

*“We’re not the classic undertaker, who just sells some goods and disposes of the dead body nicely, but we want to bring back to people what used to be normal, when it was in the hands of the family to say goodbye.” (B - Undertaker)*

### Missing inter- and intra-institutional cooperation

We found no structured cooperation pathways in the field. Collaboration at all levels depended on personal experiences and relationships of individual professionals. Inter-institutional communication and cooperation proved to be scarce. In some cases, there was no cooperation at all, which made coordinated care more difficult.

*“When the diagnosis is made, maybe someone from the pediatricians is brought in or then the parents have a one-on-one discussion with the pediatrician about what will happen and how, but not that six parties sit down at a table and discuss the case, unfortunately not.” (H3 - midwife)*

Perinatal palliative care requires comprehensive care including medical, psychosocial, and spiritual issues. However, areas of responsibility and tasks were not always clearly assigned to individual professions or institutions. Sometimes, this led to competition and frustration.

*“And of course the old conflicts between nurses and doctors come to light because there is such a high interface of things. (. . .) There is no question about it, of course, there is also competition. But we can talk about it, that’s already quite a lot.” (S - chaplain)*

Almost all interviewees pointed out that opportunities for exchange between professionals were missing. Networking events, for example, roundtable discussions, workshops, coffee chats, and conferences about perinatal palliative care were desired, especially on an interdisciplinary basis. This could contribute to better collaboration and more region-wide cooperation.

*“That’s why I think it would be good if there was first of all the possibility to get in contact with others and to say, hey, I’m interested [in caring for these families], (. . .) how do we manage it well? Joint training, perhaps, a round table every now (. . .) or case discussions or something, exchange” (H1 - midwife).*

### Perception of families

Participants vividly recalled their encounters with affected families. Parents who decided to carry on with their pregnancies were described as strong, well informed, and spiritually rooted. Participants also highlighted the cohesive bonds within these families. Interviewees had the impression that it was the parents who set the course.

*“And the families that I’ve met so far [who carried on], they made a very strong impression, I feel like they had reflected on it a lot and so I had the feeling that you were allowed to get on board with it rather than having to steer the boat.” (N1 - neonatologist)*

Participants perceived that these parents preferred a tailored form of care that addressed the tragedy of the situation and at the same time acknowledged the beautiful aspects of parenting. Professionals made high personal commitments to provide suitable care. Some of them even worked for free, handed out their private telephone numbers, and showed a flexible approach toward regulations.

*“I give my cell phone number, [and say] we can talk anytime, sometimes I say you can call me anytime, but in any case I’ll get back to you tomorrow or after the weekend. . .” (PD1 - prenatal diagnostician)*

Most psychosocial professionals thought that they had received an adequate training, where they learned to provide emotional care and orientation, to mediate relationships and to support parenting. In contrast, medical professionals felt unprepared for such situations. They acquired psychosocial skills from psychosocial colleagues and emphasized the importance of senior mentors.

*“I believe that it is very important that the senior physician takes the resident by the hand. (. . .) Because it is always very difficult to know what to say to people. That I also copied a bit from Mrs. F. (chaplain). I think that’s the real learning. That you learn how to lead conversations in such situations. That would be important.” (G1 - obstetric gynecologist)*

All interviewees described gender-specific ways of dealing with the situation. In particular, there were concerns about the availability of sufficient support options for men, especially considering the overall paucity of male caretakers in this field in Germany.

*“There are only female doctors in obstetrics, there are only female midwives and female nurses (. . .) And a male element, so to speak, especially if the fathers are to be involved, that wouldn’t be bad, but it just does not exist.” (PD3 - prenatal diagnostician)*

### Collateral beauty: Personal experiences and coping strategies of care professionals

Perinatal palliative care confronted participants with existential issues and their own value system. In some cases, a conflict of values arose during the caretaking. To be able to cope with these challenges, many professionals acquired additional qualifications and sought the support of a counselor. This was sometimes paid by private means.

*“This topic of death and grief is of course also something where we [caregivers], when we go deeper into it, where we simply have to deal with our own history, (. . .) That is why it is actually so important that we first achieve clarity for ourselves, what we want, how we deal with death and our own fears, before we even have the strength and energy to sustain others.” (H3 - midwife)*

Professionals found themselves in a borderline situation, in which they had to find a balance between authentic sympathy and professional distance. Herein, they had to avoid overload and role conflicts.

*“You have to give a lot of yourself, but not too much (. . .) As hard as it is, it’s only a job at the end of the day. And it’s often difficult. (. . .) And that’s also a role that you have to play, versus, you also have to be authentic. And that’s a very, very fine line, where you also sometimes fail, so sometimes you’re too personal, sometimes you’re then too much in the role. (. . .) It’s definitely a challenge.” (H2 - midwife)*

Interviewees reported that their abilities to deal with such situations increased over time due to professional experience and age.

*“At some point I realized that I got far too involved in the processes of the families and could no longer separate what is mine and what is theirs. When I realized that, I then did a little work on myself, and I also realize that it gets a lot easier the older I get.” (H1-midwife)*

Participants highly valued team exchange and support. They particularly appreciated the possibility of handing over their care duties to other colleagues in case of feeling overwhelmed by the situation. Team members often developed special bonds to each other. Many participants considered their team as one of the few groups of people to whom they could talk about their job experience.

*“[What helps is] To take care of yourself (. . .) and also hopefully to be in such good contact with your colleagues that you can also tell each other when it’s too much, to say, ‘You know, I can’t do that today. Could you take over?’” (S - chaplain)*

*“You have to have experienced it to understand it. So no one really understands, except someone who has cared for this, how beautiful this can be, but also how exhausting this is psychologically.” (H2 - midwife)*

Individual and team supervision were seen as valuable tools. However, there were few possibilities of supervision for medical care providers whereas psychosocial professionals had regular access to supervision.

*“Especially when situations in the delivery room are too emotional, too burdensome, those who are present there, midwifes as well as physicians, need to talk.” (H3 – midwife)*

Unexpectedly, almost all interviewees enjoyed caring for families in perinatal palliative care situations. All participants were able to identify positive aspects of their work. They perceived perinatal palliative care to be a meaningful task and reported a learning effect, which helped them to better cope with personal losses.

*“They [parents] very often say, ‘Before I met you, I had a completely different idea about death and how to cope with it. You changed my life’ (. . .) This gives me goose bumps. Because, I think, it is unbelievable, what a responsibility and what an awesome job we have. That is what others do not [understand]. ‘You are always surrounded by sadness’ [they say]. ‘No’, I say. (. . .) Okay, of course, sadness is part of it (. . .) But that there is also joy and laughter (. . .). For many, this is inconceivable.” (B - undertaker)*

*“So I learn through the care, how people deal with crises, how does life go on [after such a tragedy]. What sustains you through crises, what doesn’t? I think I’ve just become much more open. I find it totally fascinating where people draw strength from, how couples get through things. It’s a privilege to accompany it.” (PD1 - prenatal diagnostician)*

All interviewees described an increased sense of humbleness toward life and gratitude for the positive things in their lives. They experienced professional and personal growth due to their job experience. This is what participants pointed out as the “collateral beauty” of perinatal palliative care.

*You shouldn’t say that it is a positive thing when a child dies – because it is not, but there is some collateral beauty. There is something positive in all this tragedy if you know what I mean. (H2 - midwife* ***)***

On the other hand, there was the risk of becoming dependent on the gratitude of families and the feeling of being needed. This made peer support, supervision, and training particularly important.

*“The job gives back a lot. It can be such a . . . such a kick! There is a danger of becoming addicted.” (S - chaplain)*

## Discussion

### Main findings

Our findings show that care providers face insufficient structures and poor cooperation, while making an immense and partly unpaid effort for parents. Caring for affected families is an emotionally extreme situation, which raises existential questions. Nevertheless, care providers describe their work as highly rewarding for their professional and personal life. Our participants referred to this as the “collateral beauty” of their work.

### What this study adds and implicates?

While previous studies emphasize the burden of providing care for parents continuing with pregnancy after learning about the prenatal diagnosis of their unborn child.^24,29,38^ our participants prioritize the benefits of caregiving. One participant even worried about the possibility that caring for affected parents might be misused for nurturing one’s own self-esteem, which in turn could have a negative effect on parents. On the other hand, interviewees highlighted that it is sometimes difficult to simultaneously express empathy and maintain a professional attitude. These observations are consistent with studies describing personal and professional growth among professionals providing care for dying patients.^25,39–41^

Caring for affected parents motivated care professionals to reflect upon existential topics. Sometimes, conflicts of values arose.^20,23^ Participants sought the support of counselors or supervisors to help them to resolve inner conflicts and to reflect about professional boundaries and overlapping roles. So far, only few investigations have discussed the need of reflective learning, which is fundamental to achieve professional growth.^39,41,42^

Previous studies about perinatal palliative care in Bavaria indicated that there is no structured perinatal palliative care program available and no structured pathways exist between institutions providing care for affected families.^10,33^ Fragmented care has been shown to increase parental dissatisfaction.^10,16,33,43,44^ Our findings emphasize that the missing inter-institutional collaboration and professional exchange not only affects parents but also care professionals. According to our data, precarious inter-institutional structures lead professionals to bridge the gap by making immense and sometimes unpaid efforts to support the parents. Hence, they wish for a structured perinatal palliative care program to improve the provision of care and help to relieve the care professionals’ workload.

According to Morgans and Shapira^45^ professional exchange might foster peer support, insight, and a culture of compassionate collaboration. A good team spirit might act as a buffer and help to share the load between different shoulders^20,24,38,39,45^ Previous studies highlight the importance of debriefing for care professionals facing extreme situations.^9,20,23,46^

All participants demanded better access to training opportunities in perinatal palliative care. In particular, physicians and midwives felt poorly trained for difficult conversations with parents, consistent with previous research.^8,19,39^ An extensive body of literature has shown the importance of enhanced communication training.^8,21,47^ Although tailored communication strategies have been developed.^19,48,49^ our data suggest that their implementation in practice is below expectations. Our study suggests that care providers mainly learn by observing their psychosocial colleagues in multidisciplinary teams, which underscores the importance of interdisciplinary cooperation and exchange in perinatal palliative care.

This study provides an insight into experiences and needs of care providers dealing with families continuing pregnancy after learning about a severe life-limiting condition in their unborn. Professionals experience care for affected parents as challenging and demanding but also as highly rewarding. Care professionals need peer support and structures that support self-reflection and help to master ethical, professional, and personal challenges arising during the provision of care. Care professionals also emphasized their need of inter-institutional as well as inter-disciplinary exchange.

In our study area, inter-institutional collaboration and structured pathways for perinatal palliative care are still missing.^10,33^ The development and implementation of a structured perinatal palliative care program requires the promotion of professional networks and the establishment of structured inter-institutional pathways. A structured program also needs to support peer exchange and regular supervisions for all involved care providers and should provide a platform for academic exchange about best practices in perinatal palliative care. Finally, we believe that perinatal palliative care programs should be also established at tertiary medical centers in conjunction with pediatric palliative care programs, in order to advance the field through research and teaching.^50^

### Limitations

Our results stem from qualitative research and therefore seek to explore and understand the subjective perspective of individuals and groups and to develop hypotheses about a particular phenomenon.^51,52^ The unequal gender distribution (16/18 females) can be explained by the fact that the majority of professionals working in obstetrics and in the psychosocial field are female. Importantly, our sampling relied on the contacts of the network of professionals of Munich and surroundings dealing with early death, which might have led to selection bias regarding the attitude toward perinatal palliative care and the participants’ values.

## Conclusion

Due to lacking health care structures, care providers make an immense and partly unpaid effort to support affected parents. Providers learn from the encounters with parents and experience “collateral beauty,” which supports the development of a humble attitude and feelings of gratitude toward life, the feeling of having a meaningful task and professional as well as personal growth.

Requirements for a structured perinatal palliative care program are fostering peer support, ensuring regular supervision, enhancing interdisciplinary exchange, and providing opportunities for training and discussing best practices.

## Supplemental Material

sj-pdf-1-pmj-10.1177_02692163241255509 – Supplemental material for “Collateral beauty.” Experiences and needs of professionals caring for parents continuing pregnancy after a life-limiting prenatal diagnosis: A grounded theory study Supplemental material, sj-pdf-1-pmj-10.1177_02692163241255509 for “Collateral beauty.” Experiences and needs of professionals caring for parents continuing pregnancy after a life-limiting prenatal diagnosis: A grounded theory study by Konstanze Wiesner, Kerstin Hein, Gian Domenico Borasio and Monika Führer in Palliative Medicine
