# Supplementary material for: “Collateral beauty.” Experiences and needs of professionals caring for parents continuing pregnancy after a life-limiting prenatal diagnosis: A grounded theory study
Source: Palliat Med. 2024 May 30;38(6):679–88. doi: 10.1177/02692163241255509 (PMC11157982; doi:10.1177/02692163241255509)
Supplement: sj-pdf-1-pmj-10.1177_02692163241255509 – Supplemental material for “Collateral beauty.” Experiences and needs of professionals caring for parents continuing pregnancy after a life-limiting prenatal diagnosis: A grounded theory study [file sj-pdf-1-pmj-10.1177_02692163241255509.pdf]

	Interview guideline - perinatal palliative care project: Expert - Interview	
Main-questions	Sub-questions	Aim´s
**What experiences have you encountered in your professional setting while supporting families who opt to proceed with their pregnancy despite their unborn child having a life-limiting condition?**	• How did you experience care before, during, and after diagnosis/birth/death of the child?<br>• How did you perceive the families?<br>• What needs and wishes do the parents express?<br>• How did you experience communication and collaboration with parents?<br>• Were there critical moments in the care?<br>• How did you experience making the diagnosis?<br>• How did you experience the decision-making process?<br>• What constitutes successful care for you?	• Introduction/Getting to Know Each Other<br>• Experiences with Families<br>• Experiences with Perinatal Palliative Care (PPC)<br>• Approaches<br>• Attitudes/Views
**Could you tell me about the general conditions of your work?**	• How do the care structures look like?<br>• How do you handle care within these structures?<br>• What resources or support options are available to you?<br>• In your opinion, would you wish additional structures and support options? How would they look like?<br>• What role does self-care play for you?	• Experiences, Expectations, and Perceptions of Care Structures<br>• Resources<br>• Needs<br>• Role perception<br>• Implementation of Self-care
**How would you describe your experience collaborating with other professionals and institutions?**	• With whom do you collaborate?<br>• What is going well, and what is not?<br>• What opportunities does the collaboration offer?<br>• Are there challenges, and what do they look like?<br>• Where do you see room for improvement?<br>• What else is needed?	• Experiences with and expectations for collaborating with others, as well as desires regarding this cooperation.<br>• Determining the professionals involved
**Is there anything else you would like to add?**		Closing
